# Reduced right/left ventricular blood pool T2-ratio predicts congestive heart failure after STEMI

**DOI:** 10.1007/s00392-026-02868-9

**Published:** 2026-02-17

**Authors:** Felix Troger, Mathias Pamminger, Christina Tiller, Magdalena Holzknecht, Ivan Lechner, Alex Kaser, Philip Lungenschmid, Ramona Popa, Fritz Oberhollenzer, Martin Reindl, Bernhard Metzler, Sebastian J. Reinstadler, Agnes Mayr

**Affiliations:** 1 University Clinic of Radiology, Medical University of Innsbruck, Anichstrasse 35, 6020 Innsbruck, Austria; 2 University Clinic of Internal Medicine III, Cardiology and Angiology, Medical University of Innsbruck, Anichstrasse 35, 6020 Innsbruck, Austria; 3 Department of Radiology and Medical Imaging, Clinical Emergency County Hospital of Brașov, Bulevardul 15 Noiembrie 60, 500326 Brașov, Romania; 4 Faculty of Medicine, “Transilvania” University of Brașov, Nicolae Bălcescu 56, 500019 Brașov, Romania

**Keywords:** Cardiac magnetic resonance imaging, Congestive heart failure, Myocardial infarction, T2-mapping

## Abstract

**Background:**

T2-mapping of the blood-pool in cardiac magnetic resonance imaging (CMR) provides important information on blood-oxygenation, and differences between right and left ventricular (RV/LV) T2-relaxation times are linked to exercise capacity in heart failure. However, there are no data available on RV/LV T2-ratio after ST-segment elevation myocardial infarction (STEMI). Our aim was to investigate the prognostic value of RV/LV T2-ratio for the development of newly diagnosed congestive heart failure (CHF) post-STEMI.

**Methods:**

Six hundred four patients were enrolled after revascularized first-time STEMI; all patients underwent CMR within four days afterwards (interquartile range (IQR) 2–5). T2 relaxation times were measured in the RV and LV blood pool on short-axis T2-maps; T2-ratio was calculated as T2_RV_/T2_LV_. Telephonic follow-ups were performed at a median observation interval of 3.0 years. CHF was defined as cardiac decompensation symptoms requiring i.v. diuretics.

**Results:**

Median T2-ratio was 73% (IQR 65–80) and significantly lower in patients with newly diagnosed CHF (69% vs. 73%, *p* = 0.019). Dichotomized at 60% (10th percentile), patients with a reduced T2-ratio experienced CHF significantly more often (19% vs. 6%, *p* < 0.001) and sooner (55 vs. 485 days, *p* < 0.001) and were significantly older, had larger infarcts, higher peak troponin T, N-terminal pro-brain natriuretic peptide (NT-proBNP), lower LV-/RV-ejection fraction, and more commonly microvascular injuries (all *p* < 0.05). In logistic regression, T2-ratio < 60% emerged as an independent prognostic marker in multiparametric models including classic CHF risk factors. Addition of RV/LV T2-ratio to NT-proBNP resulted in a net reclassification improvement of 0.32 (95% CI 0.06–0.57, *p* = 0.016).

**Conclusion:**

CMR-derived RV/LV T2-ratio is an easily applicable tool bearing prognostic potential for CHF after STEMI.

**Graphical Abstract:**

In patients with acute revascularized STEMI, the ventricular blood pool T2-ratio was shown to be an independent prognostic marker of congestive heart failure in the aftermath of the initial hospitalization. CHF: congestive heart failure, LV: left ventricular, RV: right ventricular, PCI: percutaneous coronary intervention, STEMI: ST-elevation myocardial infarction.

**Figure:**
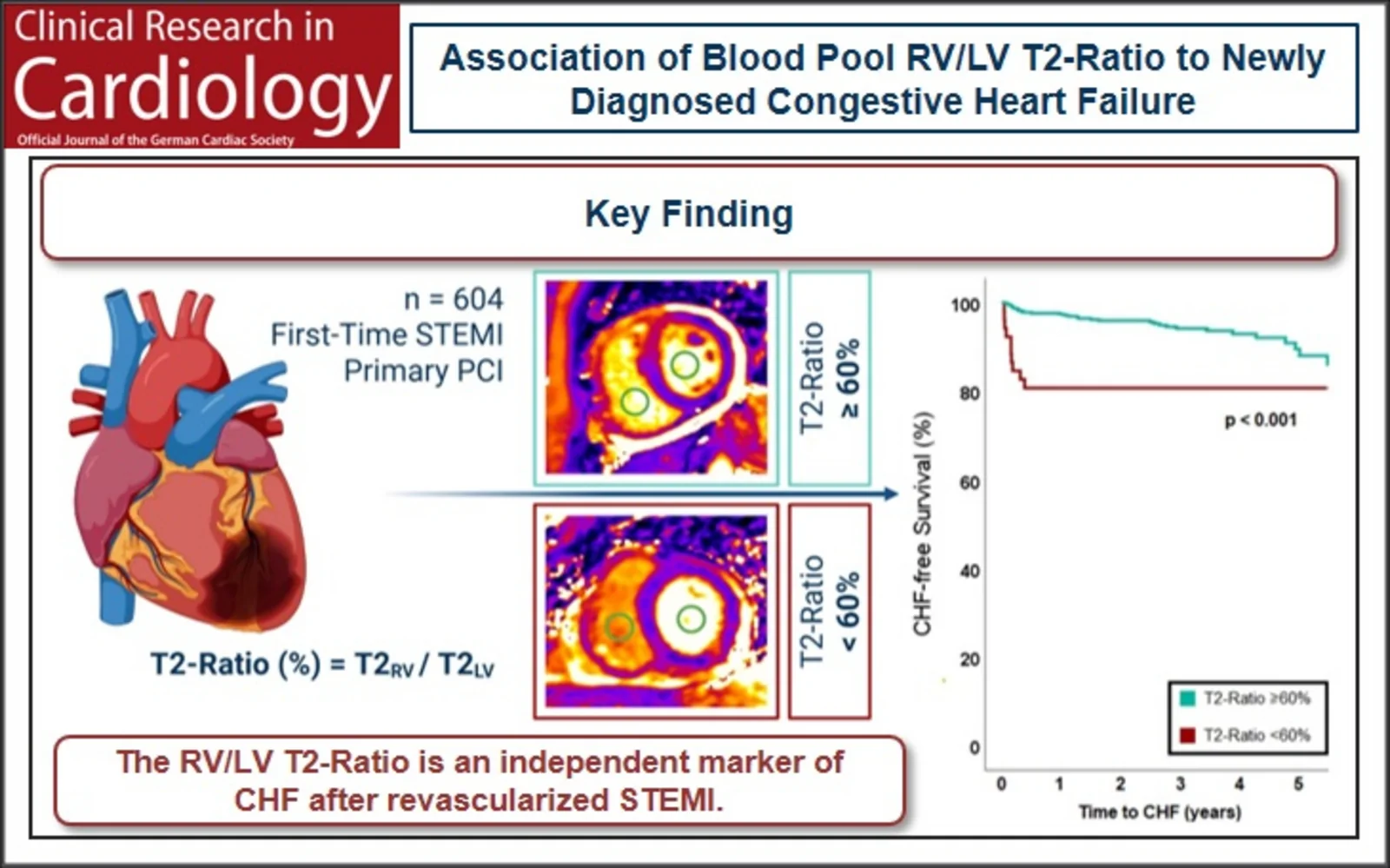

**Supplementary Information:**

The online version contains supplementary material available at https://doi.org/10.1007/s00392-026-02868-9.

## Introduction

Congestive heart failure (CHF) is a common complication after myocardial infarction, affecting approximately 15% of patients within the first year after ST-segment elevation myocardial infarction (STEMI) and having a substantial impact on quality of life and survival [1–3]. Currently, data on prognostic parameters concerning CHF after STEMI are quite scarce, with its most commonly named associated factors being increasing age, female sex, diabetes, and, in terms of imaging parameters, larger infarct size, microvascular obstruction (MVO), and intramyocardial hemorrhage (IMH) [1, 4, 5].

Recently, a ratio of T2-measurements of the right and left ventricular (RV and LV, respectively) blood pool has been introduced, correlating with exercise capacity in chronic heart failure; thereby, low T2-ratios were associated with post-exertion dyspnea and poor performance in the six-minute walk test [6]. Another study by that same research group investigated RV/LV T2-ratio to detect or exclude left-to-right shunts, with high T2-ratios indicating the presence of a shunt [7]. Overall, the proposed RV/LV T2-ratio has not yet been further investigated in myocardial infarction. However, especially its link to a worse outcome in CHF prompts the question of whether low T2-ratios are of prognostic interest in patients often being secondarily affected by CHF, such as STEMI patients.

Thus, the aims of our study were as follows: (a) to measure the ventricular blood pool RV/LV T2-ratio in a cohort of first-time STEMI patients via cardiac magnetic resonance imaging (CMR); (b) to assess its association to major adverse cardiac events (MACE), especially CHF, in this cohort; and (c) to evaluate its prognostic value in terms of CHF after STEMI.

## Methods

### Study population

Patients were included from the MARINA-STEMI (Magnetic Resonance Imaging In Acute ST-Elevation Myocardial Infarction) study (NCT04113356).

Inclusion criteria included (a) first-time STEMI (according to the European Society of Cardiology guidelines [8]) and (b) treated by primary percutaneous coronary intervention (PCI) within 24 h following symptom onset.

Exclusion criteria comprised (a) age < 18 years, (b) an estimated glomerular filtration rate < 30 ml/min/1.73 m^2^, (c) a Killip class ≥ 3 at the time of CMR imaging, (d) any history of a previous myocardial infarction or coronary intervention, and (e) any CMR contraindication (pacemaker, orbital foreign body, cerebral aneurysm clip, manifest claustrophobia, and known or suggested hypersensitivity reactions to gadolinium-based contrast media).

This study was approved by the local Research Ethics Committee and conforms to the Declaration of Helsinki. No sex-based or race/ethnicity-based differences were present.

### Cardiovascular magnetic resonance imaging

All CMR scans were performed on a 1.5 Tesla MAGNETOM AvantoFit scanner (Siemens, Erlangen, Germany) within the first week after PCI. Regarding image acquisition and post-processing, our standardized research protocol has been applied [9].

To sum up, functional-volumetric assessment was performed using short‐axis cine images acquired by ECG‐triggered balanced steady‐state free precession bright‐blood sequences. Standard software (Circle Cardiovascular Imaging: cvi42, Calgary, Canada) was used for post-processing analyses with semi-automatic detection of LV endo- and epicardial borders; papillary muscles were excluded from myocardial mass and included in the LV volume. End-diastolic volume and end-systolic volume were then divided by the body surface area (computed via the Du Bois formula [10]) to obtain indexed values (EDVi and ESVi, respectively). Late gadolinium‐enhanced imaging was conducted by administering 0.2 mmol/kg of Gd-DO3A-butrol (Gadovist®, Bayer Vital, Leverkusen, Germany) and obtaining images 15 min thereafter using ECG‐triggered phase‐sensitive inversion recovery sequences. The infarcted area was defined by hyperenhancement at a threshold of + 5 standard deviations above the signal intensity of remote myocardial tissue of the diametrically opposite LV myocardium. Infarct size was expressed as absolute infarct mass (in g, as a product of cumulative infarct area in the affected slices, slice thickness including interslice gap, and specific myocardial mass) as well as the percentage of LV myocardial mass yielded in volumetric measurements. MVO was defined as hypoenhancement within the infarct area on late gadolinium enhanced images. To assess IMH, T2*-mapping was applied using a breath‐hold, cardiac‐gated gradient echo sequence with 8 echoes obtained in 3 matching short‐axis slices before contrast agent administration. Motion correction was applied to reduce corresponding artifacts. IMH was defined as the region within the infarcted area with average T2* values below 20 ms [11].

**Fig. 1:**
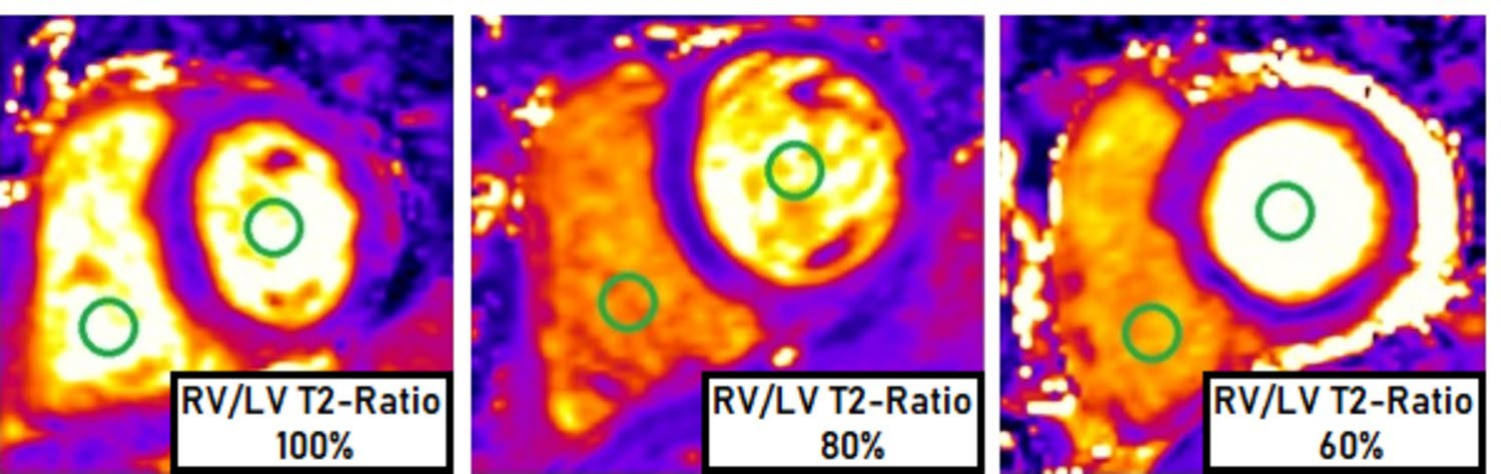
Short-axis T2-maps of different STEMI patients depicting the determination of the ventricular blood pool RV/LV T2-ratio. LV: left ventricular, RV: right ventricular, STEMI: ST-segment elevation myocardial infarction

For the T2-maps, three single-shot short-axis images were acquired at different T2-preparation times (0 ms, 25 ms, and 55 ms, respectively), and imaging parameters were: echo time = 1 ms; flip angle = 70°; repetition time = 3 × R-R interval; acquisition matrix 112 × 192; slice thickness 8 mm; field of view adjusted as per subject size. Motion correction and fitting were performed to estimate coefficients of decay function, which were then used to display the T2 times. An in-built specific color look-up table was then used to derive the colored T2 maps. For the ventricular T2 relaxation times, a region of interest was drawn into the RV and LV blood pool, respectively, with an area of at least 100mm^2^ excluding papillary muscles and avoiding obvious flow artifacts, as can be seen in Fig. 1. The T2-ratio was calculated as *100* × *T2*_*RV*_*/T2*_*LV*_ and is presented as a percentage. Further, T2-ratio values were dichotomized at the 10th percentile to split patients with the lowest T2-ratio values from those with higher values.

### Follow-up period

All patients were re-examined in the course of a clinical follow-up four and twelve months after the index hospitalization, comprising a standardized questionnaire, physical examination, blood and urine analysis, echocardiography, and ECG. In patients with the index event dating back more than 400 days at the time of the analysis, telephonic follow-ups were performed with the primary intention to ask for MACE, being defined as a combined clinical endpoint comprising all-cause death, myocardial re-infarction, and CHF. The latter was as the requirement of intravenous diuretics after discharge or any sign of heart failure necessitating hospital readmission [12, 13] and represented the primary study endpoint. For all MACE endpoints, the respective event was handled as a binary variable, with the interval between STEMI and the respective endpoint representing the time to the first event.

### Statistical analysis

SPSS Statistics 30.0.0 (IBM, Armonk, NY, USA) and R Statistical Software v4.5.1 (R Core Team, 2024) were used for statistical analyses. All results for continuous variables are expressed as medians with corresponding interquartile range (IQR), categorical variables as absolute numbers and percentages. Differences in categorical variables between two or more groups were tested by the Chi-square test. Differences in continuous variables were tested using Mann–Whitney *U* test. To evaluate the incremental prognostic benefit of decreased RV/LV T2-ratio over other risk factors for the prediction of CHF, net reclassification improvement (NRI) and integrated discrimination improvement were calculated by using the R package “PredictABEL”. Binary logistic regression was performed to evaluate independent markers for CHF after STEMI; in addition to the binary T2-ratio (dichotomized at the 10th percentile and using the Youden-index), variables of clinical interest (N-terminal pro-brain natriuretic peptide (NT-proBNP), LV ejection fraction (EF), infarct size, IMH) as well as variables with a *p*-value < 0.100 in univariable analysis were included in our multivariable models, which were composed of one clinical, laboratory and CMR parameter each. To enhance readability and interpretability, metric variables were therefore stratified into quartiles. Lastly, CHF-free survival was assessed using a Kaplan–Meier plot with log-rank testing. A *p*-value < 0.050 was considered as statistically significant. Finally, intraclass correlation coefficient (two-way) was computed to display intra- and interobserver reliability.

## Results

### Baseline patient characteristics

Overall, 604 patients were included in this current analysis. Median age was 58 years (IQR 52–67) and 114 patients (19%) were female. The total ischemia time (i.e., pain-to-balloon) was 181 min (IQR 111–315). Baseline CMR was performed 4 days after PCI (IQR 3–5). A detailed list of patient characteristics is shown in Table 1.

**Table 1:** Baseline patient characteristics

	Total (*n* = 604)	No CHF (*n* = 561)	CHF (*n* = 43)	*p*-value
Age, years	58 (52–67)	58 (52–66)	66 (57–76)	** < 0.001**
Female, *n* (%)	114 (19)	103 (18)	11 (26)	0.244
BMI, kg/m^2^	26 (24–29)	26 (24–29)	26 (24–30)	0.665
Smokers, *n* (%)	333 (55)	313 (56)	20 (47)	0.238
Diabetes, *n* (%)	50 (8)	40 (7)	10 (23)	** < 0.001**
Hypertension, *n* (%)	258 (43)	231 (41)	27 (63)	**0.006**
Hypercholesterolemia, *n* (%)	312 (52)	288 (51)	24 (56)	0.571
Total ischemia time, min	181 (111–315)	177 (111–310)	210 (122–337)	0.169
**Culprit vessel, *n* (%)**				
- LCA	3 (0.5)	3 (0.5)	0 (0)	0.636
- LAD	273 (45)	249 (44)	24 (56)	
- RCA	232 (38)	219 (39)	13 (30)	
- CX	92 (15)	86 (15)	6 (14)	
- R. intermedius	4 (1)	4 (1)	0 (0)	
**Lab data**				
- Peak creatinine, mg/dl	1.1 (1.0–1.2)	1.1 (1.0–1.2)	1.2 (0.9–1.4)	0.100
- Peak TnT, ng/ml	4645 (2098–7533)	4378 (2026–7230)	8734 (5088–17051)	** < 0.001**
- Peak CK, U/l	1859 (962–3380)	1778 (939–3266)	3713 (1823–4749)	** < 0.001**
- Peak CRP, mg/dl	2.6 (1.4–4.8)	2.5 (1.3–4.6)	4.7 (2.1–6.6)	**0.001**
- Peak NT-proBNP, ng/l	1438 (751–2624)	1372 (743–2522)	2426 (1202–6023)	** < 0.001**
**CMR Data**				
- LV-EF, %	49 (42–55)	50 (43–55)	41 (32–49)	** < 0.001**
- LV-EDVi, ml/m^2^	84 (71–95)	84 (71–95)	88 (70–102)	0.586
- LV-ESVi, ml/m^2^	42 (33–52)	42 (33–51)	47 (37–69)	**0.007**
- LV-Myo Mass, g	124 (107–144)	124 (107–143)	124 (107–149)	0.561
- GLS, %	− 11 (− 13 to − 8)	− 11 (− 13 to − 8)	− 8 (− 11 to − 6)	** < 0.001**
- RV-EF, %	51 (45–57)	51 (45–57)	49 (42–57)	0.493
- RV-EDVi, ml/m^2^	78 (66–91)	78 (67–92)	70 (58–88)	**0.016**
- RV-ESVi, ml/m^2^	38 (30–48)	38 (30–48)	34 (27–51)	0.381
- Infarct size, g	19 (9–32)	18 (8–30)	35 (19–48)	** < 0.001**
- Relative infarct size, % of Myo mass	15 (8–24)	15 (7–23)	28 (17–39)	** < 0.001**
- MVO, *n*(%)	348 (58)	316 (56)	32 (74)	**0.022**
- IMH, *n*(%)	195 (32)	165 (29)	30 (70)	** < 0.001**
- T2 LV, ms	178 (164–198)	178 (164–198)	180 (156–194)	0.455
- T2 RV, ms	131 (121–140)	132 (121–140)	121 (114–130)	** < 0.001**
- T2-ratio, %	73 (65–80)	73 (66–80)	69 (60–76)	**0.019**
- T2-ratio < 60%, n(%)	52 (9)	42 (7)	10 (23)	** < 0.001**

*CHF* congestive heart failure, *CK* creatine kinase, *CMR* cardiac magnetic resonance imaging, *CRP* C-reactive protein, *CX* circumflex artery, *EDVi* indexed end-diastolic volume, *EF* ejection fraction, *ESVi* indexed end-systolic volume, *GLS* global longitudinal strain, *IMH* intramyocardial hemorrhage, *LAD* left anterior descending artery, *LCA* left coronary artery, *LV* left ventricle/ventricular, *MVO* microvascular obstruction, *NT-proBNP* N-terminal pro-brain natriuretic peptide, *RCA* right coronary artery, *RV* right ventricle/ventricular, *TnT* troponin T

### Congestive heart failure

After a median observational interval of three years (IQR 1.2–4.5), CHF occurred in 43 patients (7%) within a median time of 142 days after the index event. Patients with CHF were significantly older and more commonly affected by diabetes and hypertension (all *p* < 0.010); furthermore, they had higher values of peak highly-sensitive troponin T, creatine kinase (CK), and NT-proBNP (all *p* ≤ 0.001). In terms of imaging parameters, patients affected by CHF had significantly lower LV-EF, higher LV-ESVi, reduced global longitudinal strain (GLS), larger infarcts (absolute and relative compared to LV myocardial mass), and more often MVO and IMH (all *p* < 0.030). A detailed comparison of patients with and without CHF is shown in Table 1.

### T2-ratio

**Fig. 2:**
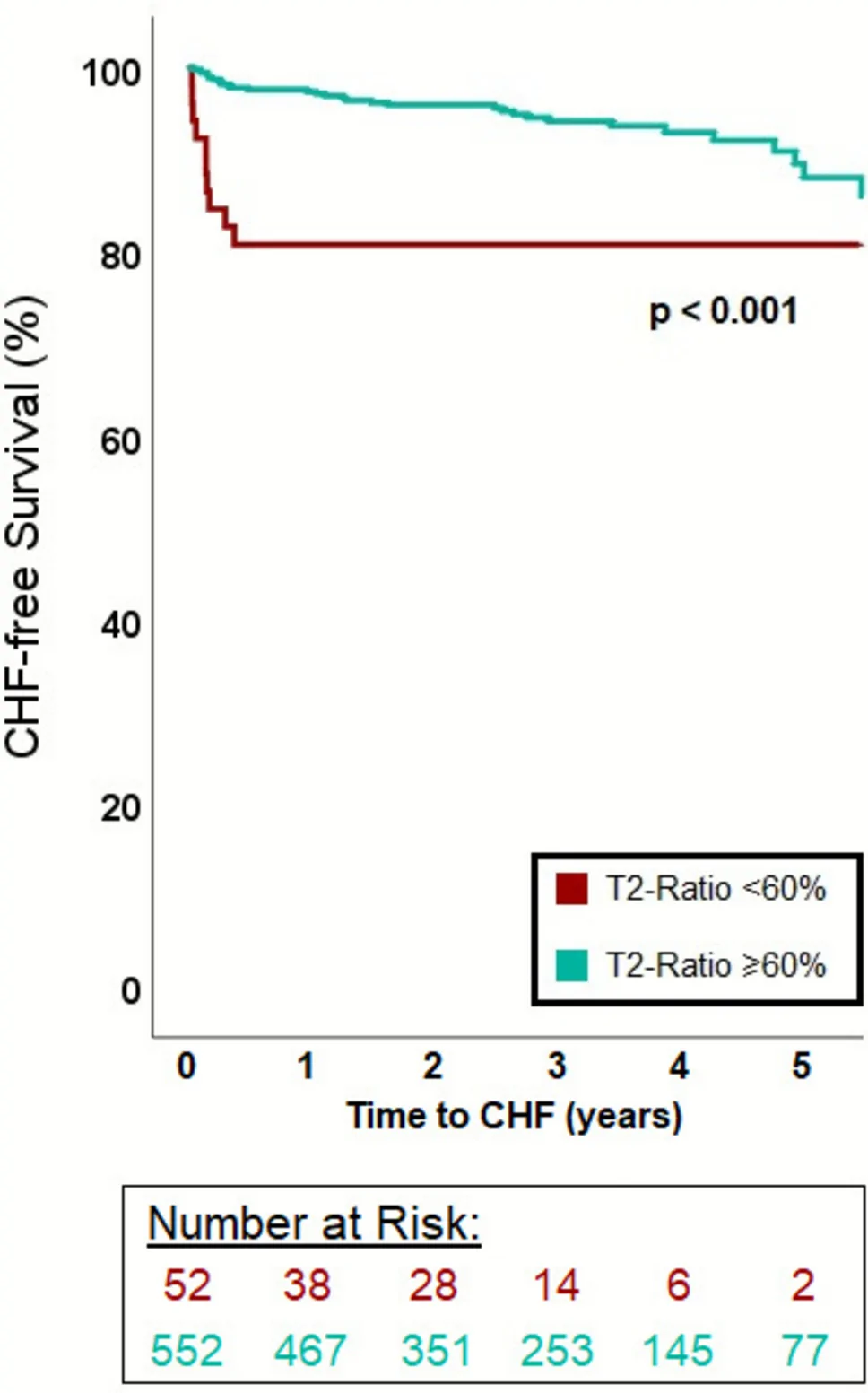
Kaplan–Meier plot depicting the CHF-free survival up to the first CHF episode in patients with a T2-ratio < 60% and ≥ 60%. CHF: congestive heart failure

Median RV/LV T2-ratio was 73% (IQR 65–80) and patients with CHF during observation interval had a significantly lower ratio (69%, IQR 60–76 vs. 73%, IQR 66–80, *p* = 0.019). The 10th percentile, which was further used to dichotomize the patient cohort, was at 60%. Patients with a T2-ratio below 60% were more commonly affected by CHF (19% vs. 6%, *p* = 0.001) and experienced CHF significantly sooner after STEMI (55 days, IQR 13–76 vs. 485 days, IQR 99–1226, *p* < 0.001). In absolute values, patients with CHF had lower RV-T2 values (*p* < 0.001) and no significant difference in LV-T2 values (*p* = 0.455). Moreover, patients with a T2-ratio below 60% were significantly older, had higher peak values of troponin T, CK, C-reactive protein (CRP) and NT-proBNP, lower LV- and RV-EF, higher LV-ESVi, reduced GLS, larger infarct size (absolute and relative) and more commonly MVO and IMH (all *p* < 0.050). A detailed comparison of patients with T2-ratio < 60% and ≥ 60% is shown in Table 2. A receiver operating characteristic curve examining different T2-ratio thresholds for CHF is shown in Fig. 2. Using the Youden-index for the dependent variable CHF, the optimized threshold was 67%, with 169 patients (28%) lying below this value. Dichotomized at this threshold there were the same significant inter-group differences seen in Table 2 as with 60%, with the exception of RV-EF (*p* = 0.073). In terms of MACE, patients with a T2-ratio below 67% suffered CHF significantly more often (12% vs. 5%, *p* = 0.005), but not the other MACE endpoints (all *p* > 0.1).

**Table 2:** Comparison of patients with T2-ratio above and below 60%

	T2-ratio ≥ 60% (*n* = 552)	T2-ratio < 60% (*n* = 52)	*p*-value
Age, years	58 (52–66)	66 (57–75)	** < 0.001**
Female, *n* (%)	102 (18)	12 (23)	0.418
BMI, kg/m^2^	26 (24–29)	25 (34–28)	0.118
Smokers, *n* (%)	306 (55)	27 (52)	0.626
Diabetes, *n* (%)	48 (9)	2 (4)	0.225
Hypertension, *n* (%)	237 (43)	21 (40)	0.722
Hypercholesterolemia, *n* (%)	285 (52)	27 (52)	0.968
Total ischemia time, min	178 (110–315)	195 (128–314)	0.419
**Culprit vessel, *n* (%)**			
- LCA	3 (0.5)	0 (0)	0.603
- LAD	250 (45)	23 (44)	0.603
- RCA	208 (38)	24 (46)	0.603
- CX	87 (16)	5 (10)	0.603
- R. intermedius	4 (1)	0 (0)	0.603
**Lab data**			
- Peak creatinine, mg/dl	1.1 (1.0–1.2)	1.2 (1.1–1.4)	** < 0.001**
- Peak TnT, ng/ml	4372 (1960–7230)	7363 (4874–12,637)	** < 0.001**
- Peak CK, U/l	1779 (919–3269)	3027 (1610–4760)	** < 0.001**
- Peak CRP, mg/dl	2.6 (1.3–4.7)	3.5 (1.6–7.4)	**0.048**
- Peak NT-proBNP, ng/l	1372 (728–2430)	1891 (1130–6007)	** < 0.001**
**CMR data**			
- LV-EF, %	49 (43–55)	42 (33–52)	** < 0.001**
- LV-EDVi, ml/m^2^	83 (71–94)	91 (74–98)	0.079
- LV-ESVi, ml/m^2^	42 (33–51)	49 (43–62)	** < 0.001**
- LV-Myo mass, g	124 (108–144)	122 (103–142)	0.526
- GLS, %	− 11 (− 13 to − 8)	− 8 (− 11 to − 7)	** < 0.001**
- RV-EF, %	51 (45–57)	49 (39–55)	**0.025**
- RV-EDVi, ml/m^2^	78 (67–92)	76 (62–91)	0.569
- RV-ESVi, ml/m^2^	38 (30–48)	40 (33–50)	0.284
- Infarct size, g	18 (8–30)	28 (12–44)	**0.003**
- Relative infarct size, % of Myo mass	15 (7–24)	23 (11–32)	** < 0.001**
- MVO, *n* (%)	311 (56)	37 (71)	**0.041**
- IMH, *n* (%)	170 (31)	25 (48)	**0.011**
- T2 LV, ms	176 (162–194)	215 (187–243)	** < 0.001**
- T2 RV, ms	132 (122–141)	115 (100–132)	** < 0.001**

*CHF* congestive heart failure, *CK* creatine kinase, *CMR* cardiac magnetic resonance imaging, *CRP* C-reactive protein, *CX* circumflex artery, *EDVi* indexed end-diastolic volume, *EF* ejection fraction, *ESVi* indexed end-systolic volume, *GLS* global longitudinal strain, *IMH* intramyocardial hemorrhage, *LAD* left anterior descending artery, *LCA* left coronary artery, *LV* left ventricle/ventricular, *MVO* microvascular obstruction, *NT-proBNP* N-terminal pro-brain natriuretic peptide, *RCA* right coronary artery, *RV* right ventricle/ventricular, *TnT* troponin T

A reduced T2-ratio < 60% was more common in MACE patients compared to those without MACE (16% vs. 8%, *p* = 0.019). There was a greater portion of patients with a decreased T2-ratio in those experiencing death of any cause (21% vs. 8%, *p* = 0.049) and of cardiovascular cause in particular (50% vs. 8%, *p* < 0.001). MACE data are shown in Table 3. For CHF, adding a reduced T2-ratio < 60% to peak NT-proBNP, diabetes, IMH or relative infarct size resulted in a significant continuous NRI (0.1, 0.2) of 0.315 each (95% confidence interval (CI) 0.059–0.572, *p* = 0.016). Furthermore, the integrated discrimination improvement was significant when adding T2-ratio to these parameters (NT-proBNP: 0.01, 95% CI 0.00–0.03, *p* = 0.038; diabetes: 0.02, 95% CI 0.00–0.05, *p* = 0.022; IMH: 0.02, 95% CI 0.00–0.05, *p* = 0.033; relative infarct size: 0.02, 95% CI 0.00–0.04, *p* = 0.031). Compared to absolute T2-values of the respective ventricular blood pool, the T2-ratio showed a significant continuous NRI for both the RV (NRI: 0.305, 95% CI 0.048–0.561, *p* = 0.020) and LV (NRI: 0.411, 95% CI 0.115–0.708, *p* = 0.007). Results of multivariable logistic regression analyses using these absolute values is shown in the supplement. There were no sex-specific differences concerning MACE, T2-values and T2-ratio as well as other relevant CMR findings (all *p* > 0.050). Intra- and interobserver reliability were both in good agreement for T2-ratio (intraclass correlation coefficients of 0.80 (95% CI 0.71–0.87) and 0.71 (95% CI 0.57–0.80), respectively.

**Table 3:** Overview on MACE data

	Total (*n* = 604)	T2-ratio ≥ 60% (*n* = 552)	T2-ratio < 60% (*n* = 52)	*p*-value
**MACE, *n* (%)**	77 (13)	65 (12)	12 (23)	**0.019**
- All-cause death	19 (3)	15 (3)	4 (8)	**0.049**
- CV death	8 (1)	4 (1)	4 (8)	** < 0.001**
- Re-infarction	29 (5)	25 (5)	4 (8)	0.308
- CHF	43 (7)	33 (6)	10 (19)	** < 0.001**

*CHF* congestive heart failure, *CV* cardiovascular, *MACE* major adverse cardiac events

### Logistic regression analysis and CHF-free survival

**Fig. 3:**
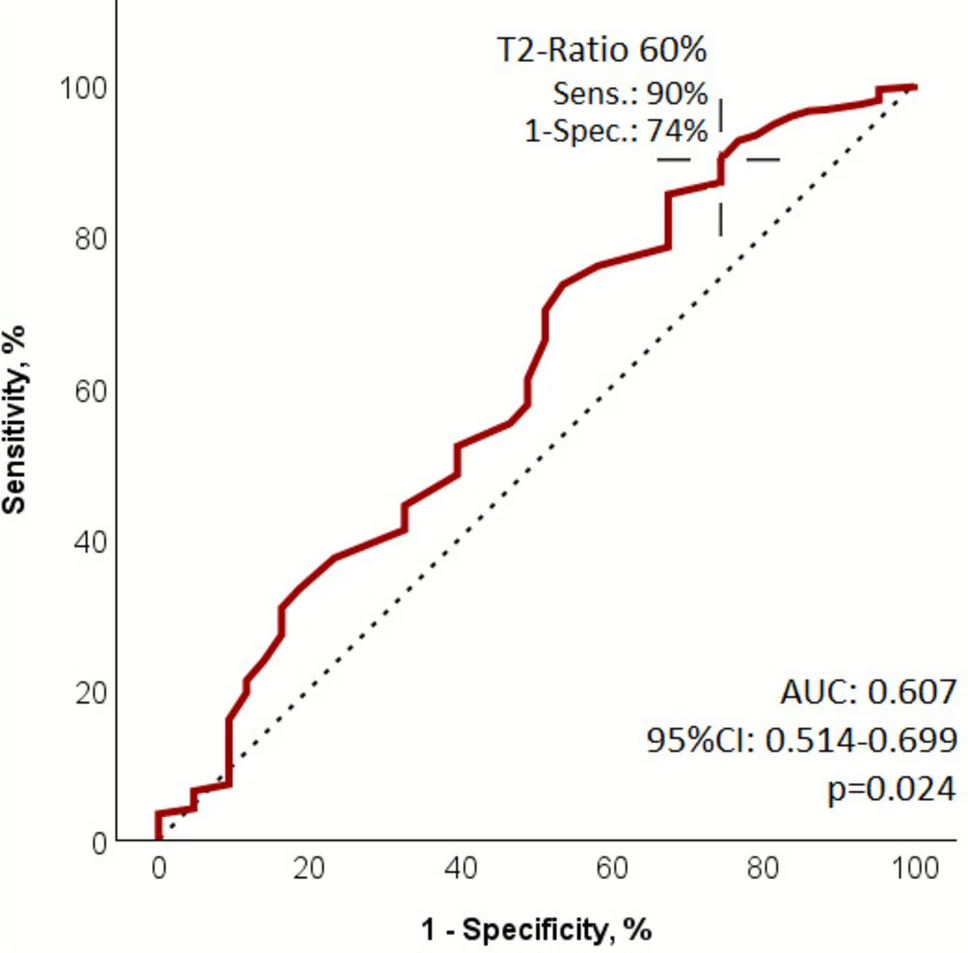
Receiver operating characteristic curve displaying sensitivity and (1-specificity) of T2-ratio at different cutoffs, with 60% being used as primary threshold in this study. AUC: area under the curve, CI: confidence interval

**Fig. 4:**
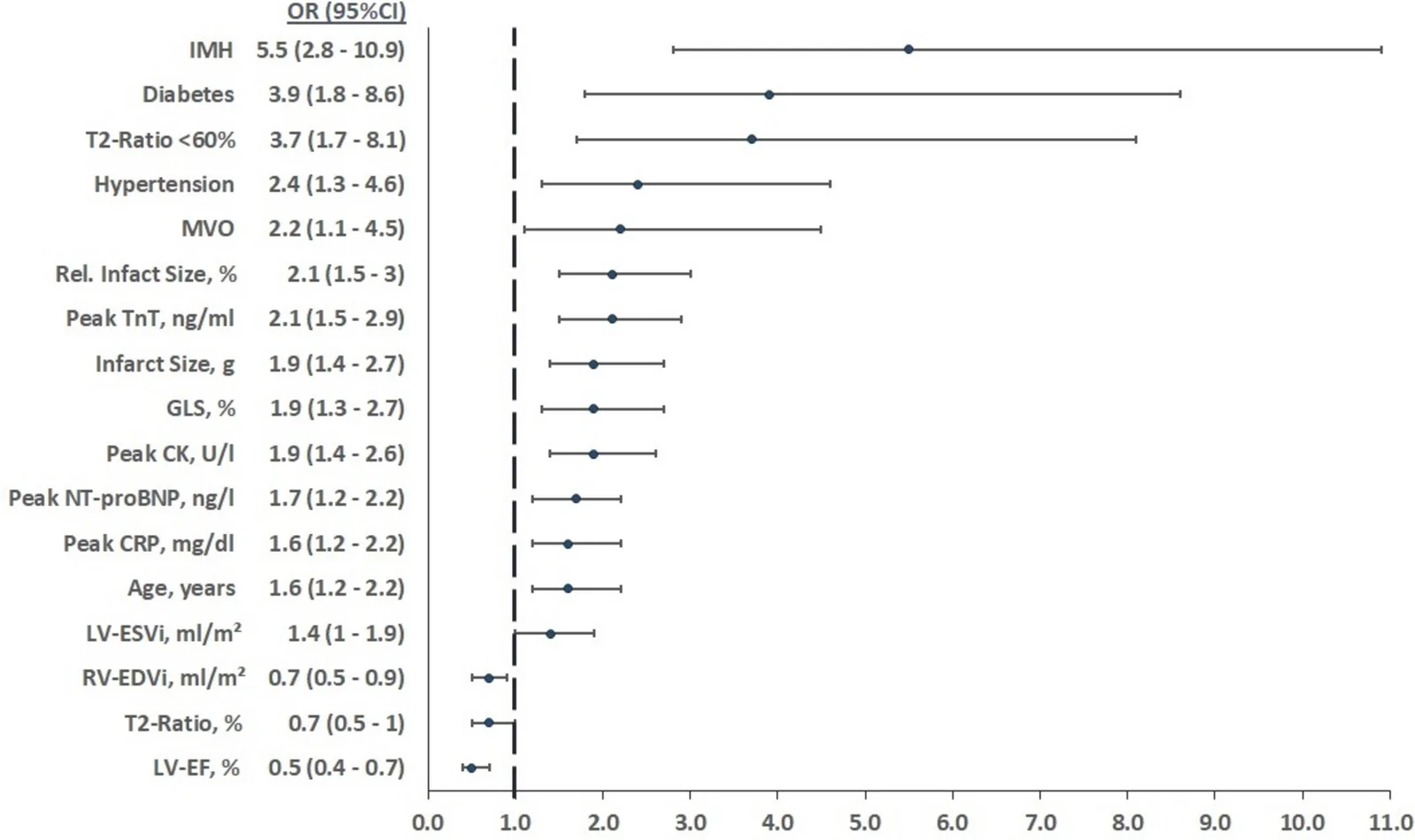
Forest plot depicting the results of univariable analysis, sorted by odds ratio in descending order. CI: confidence interval, CK: creatine kinase, CRP: C-reactive protein, EDVi: indexed end-diastolic volume, ESVi: indexed end-systolic volume, EF: ejection fraction, GLS: global longitudinal strain, IMH: intramyocardial hematoma, LV: left ventricular, MVO: microvascular obstruction, NT-proBNP: N-terminal pro-brain natriuretic peptide, OR: odds ratio, RV: right ventricular, TnT: troponin T

In univariable analyses, RV/LV T2-ratio was significantly predictive for CHF (odds ratio (OR) 0.7, 95% CI 0.5–1.0, *p* = 0.039), as was dichotomized T2-ratio < 60% (OR 3.7, 95% CI 1.7–8.1, *p* < 0.001). A T2-ratio below the 10th percentile was significantly linked to CHF in several multivariable models including: diabetes, peak NT-proBNP and IMH (model 1); diabetes, peak NT-proBNP and relative infarct size (model 2); as well as age, diabetes and IMH (for T2-ratio < 60%: OR 2.8, 95% CI 1.2–6.5, *p* = 0.019); hypertension and GLS (for T2-ratio < 60%: OR 2.7, 95% CI 1.1–6.5, *p* = 0.030); diabetes, hypertension and peak CK (T2-ratio < 60%: OR 3.4, 95% CI 1.5–7.8, *p* = 0.005); diabetes, peak CRP and relative infarct size (T2-ratio < 60%: OR 3.3, 95% CI 1.4–7.5, *p* = 0.005). The results of uni- and multivariable binary logistic regression analysis are listed in Table 4. In addition, the results of univariable analysis are shown in Fig. 3. T2-ratio below 67% (Youden-index) was significant in univariable analysis (OR 2.4, 95% CI 1.3–4.5, *p* = 0.006), but not in multivariable models when replacing 60% as the threshold. In Kaplan–Meier analysis, patients with RV/LV T2-ratio < 60% had a significantly shorter mean CHF-free survival (1589 ± 104 days vs. 2702 ± 44 days, *p* < 0.001). The corresponding Kaplan–Meier plot is shown in Fig. 4. When choosing a cut-off at two years, patients with a T2-ratio > 60% still had a significantly shorter survival time (591 vs. 697 days, *p* < 0.001), although 13 CHF events (30%) fell out of analysis. By widening the observation window to three years, survival time was also significantly shorter (895 vs. 1059 days, *p* < 0.001), with 9 events having been excluded (21%).

**Table 4:** Results of logistic regression analysis. Metric variables were stratified into quartiles

Variable(s)	Odds ratio	95% confidence interval	*p*-value
** > Univariable analyses**			
T2-ratio, %	0.7	0.5–1.0	**0.039**
T2 LV, ms	0.9	0.7–1.2	0.371
T2 RV, ms	0.5	0.4–0.8	** < 0.001**
T2-ratio < 60%	3.7	1.7–8.1	** < 0.001**
Age, years	1.6	1.2–2.2	**0.001**
Diabetes	3.9	1.8–8.6	** < 0.001**
Hypertension	2.4	1.3–4.6	**0.007**
Peak TnT, ng/ml	2.1	1.5–2.9	** < 0.001**
Peak CK, U/l	1.9	1.4–2.6	** < 0.001**
Peak CRP, mg/dl	1.6	1.2–2.2	**0.002**
Peak NT-proBNP, ng/l	1.7	1.2–2.2	**0.001**
LV-EF, %	0.5	0.4–0.7	** < 0.001**
LV-ESVi, ml/m^2^	1.4	1.0–1.9	**0.029**
GLS, %	1.9	1.3–2.7	** < 0.001**
RV-EDVi, ml/m^2^	0.7	0.5–0.9	**0.020**
Infarct size, g	1.9	1.4–2.7	** < 0.001**
Relative infarct size, % of Myo mass	2.1	1.5–3.0	** < 0.001**
MVO	2.2	1.1–4.5	**0.025**
IMH	5.5	2.8–10.9	** < 0.001**
** > Multivariable analyses**			
*- Model 1*			
T2-ratio < 60%	3.0	1.7–7.2	**0.013**
Diabetes	4.5	1.9–10.5	** < 0.001**
Peak NT-proBNP, ng/l	1.2	0.9–1.7	0.207
IMH	4.5	2.2–9.3	** < 0.001**
*- Model 2*			
T2-ratio < 60%	3.0	1.3–7.1	**0.011**
Diabetes	4.8	2.0–11.6	** < 0.001**
Peak NT-proBNP, ng/l	1.2	0.9–1.7	0.254
Relative infarct size, % of Myo mass	2.0	1.4–2.8	** < 0.001**

*CK* creatine kinase, *CRP* C-reactive protein, *EF* ejection fraction, ESVi indexed end-systolic volume, *GLS* global longitudinal strain, *IMH* intramyocardial hemorrhage, *LV* left ventricle/ventricular, *MVO* microvascular obstruction, *NT-proBNP* N-terminal pro-brain natriuretic peptide, *RV* right ventricle/ventricular, *TnT* troponin T

## Discussion

To the best of our knowledge, this study is the first to assess the association of a ventricular blood pool T2-ratio and the development of CHF after STEMI.

This study’s results comprise the following: (a) the ventricular RV/LV T2-ratio was significantly decreased at baseline CMR in patients developing CHF after revascularized STEMI; (b) patients with a T2-ratio of < 60% (i.e. 10th percentile) were older and had significantly more severe infarctions (in terms of laboratory findings, cardiac function, infarct size, presence of MVO and IMH); (c) patients with a T2-ratio < 60% suffered MACE more frequently and experienced CHF earlier and more common than those with a higher ratio; (d) a T2-ratio of < 60% was shown to be a predictor of CHF independently of several traditional risk factors for adverse events after STEMI.

Although there are limited data on T2-measurements of the blood pool, there are currently three major studies available providing data on this method: chronologically, the first one investigated the RV/LV T2-ratio in ruling out a left-to-right shunt, whereby a ratio above 78% showed high sensitivity, specificity, and predictive value for the presence of a shunt as well as a high reproducibility, finally being superior to an increase in RV-EDVi [7]. In 2023, another study showed that the T2-ratio was significantly lower in patients with pulmonary hypertension, claiming it to be an additive prognostic marker in its severity evaluation [14]. Lastly, the most recent study investigated the association of blood pool T2-ratio and exercise capacity in chronic heart failure, showing decreased T2-ratio to be an independent marker for low performance in the six-minute walk test [6]. To sum up these current findings, a pathologically increased T2-ratio indicated left-to-right shunt, whilst a low ratio was indicative of pulmonary hypertension on one hand and a lower exercise capacity in chronic heart failure on the other hand. In concordance with our study, especially this latter study is of particular interest, with a lower T2-ratio in our study being also indicative of CHF; further, T2-ratio values in our study were well in line with this study, which provided a mean RV/LV T2-ratio of 74% in healthy individuals and, opposed to that, of 50% in patients with heart failure with reduced EF (in our study, mean T2-ratio was 73 ± 11% in patients without CHF and 69 ± 12% in patients with CHF). Beyond that, using the RV/LV T2-ratio instead of absolute blood pool T2 values, which still showed significant differences, eliminates the risk of device-specific deviations in reference values, while also providing more global information on cardiac function than single values alone. Although blood pool mapping in general is assumed to be partly dependent on serum hemoglobin and oxygenation [7, 15], hemoglobin and serum iron correlated only at a very low level with blood pool T2 values (Spearman’s rho < 0.3).

In this current study, a ventricular T2-ratio below 60% had the highest OR besides diabetes for CHF in univariable logistic regression; in multivariable analysis, it was predictive for CHF in several models containing classic risk factors such as diabetes, arterial hypertension, and NT-proBNP, which are amongst the most influential factors predisposing for CHF after myocardial infarction [1, 4, 16]. Adding reduced T2-ratio to peak NT-proBNP, diabetes, IMH, or relative infarct size resulted in a significant NRI of more than 30%. In terms of imaging parameters, a CMR/SPECT study including 1889 STEMI patients showed infarct size to be strongly correlated with hospitalization for CHF [17], a result that is very much in line with our study; even in a multivariable model also taking infarct size into account (model 2), the predictive value of a T2-ratio < 60% was of statistical significance, hinting that this easily applicable parameter could be a useful tool in prognosis estimation. Another study investigating the development of CHF after infarction in more than 1300 patients found higher CK levels as well as LAD as the culprit vessel to be the most important risk factors for CHF [18]. Whilst in our study LAD as the culprit vessel was not significantly more common in patients with CHF, peak CK was markedly different in these two groups; however, a T2-ratio < 60% was still a significant predictor of CHF in a multivariable model containing peak CK. As opposed, infarction involving the RV did not show any association with CHF or T2-ratio in our study – lastly, in a multivariable “RV model” consisting of T2-ratio < 60%, RCA as the culprit vessel, RV infarction, and RV-EDVi, T2-ratio emerged as the only significant predictor of CHF (OR 7.2, 95% CI 3.0–17.1, *p* < 0.001). Then, another decisive factor associated with CHF after myocardial infarction is increasing age [19]. Nevertheless, the present data indicate RV/LV T2-ratio to be a prognostic marker of CHF independently of age. The same accounts for diabetes, which also predisposes for CHF [20, 21], but did not overrule reduced T2-ratio in multivariable analysis. Further, concerning laboratory findings, NT-proBNP is regarded as a well-established predictor of CHF [22, 23] and represented a strong predictor in our study as well. In current literature, CRP, as a surrogate of inflammatory processes, has been described as a risk marker for CHF after STEMI [24, 25]; however, it was not a significantly predictive marker in our study’s multivariable logistic regression. In terms of absolute blood pool T2 values, T2-ratio showed a slight superiority compared to absolute values. However, the ratio itself also bears the advantage that it is a parameter independent of differences concerning vendor- or device-related properties that might influence absolute values.

Over the last few years, imaging parameters have emerged to be of incremental prognostic value in STEMI patients: besides functional parameters, infarct size and IMH are currently regarded the strongest parameters of adverse outcome after STEMI in general and the development of CHF in particular [11, 26–30]. A plethora of studies have already shown the impact of larger infarcts on outcome, e.g. the aforementioned 2016 multicenter study, that impressively demonstrated the CMR-derived infarct size’s link to all-cause mortality and hospitalization [17]. Regarding IMH, more recent studies have shown its importance in estimating prognosis after STEMI in terms of adverse outcomes [11, 31]. Both parameters have also shown important prognostic value for CHF in this current study, with IMH showing the highest OR of all examined parameters in univariable analysis.

Lastly, other factors that were reported to predispose for CHF after myocardial infarction, such as female sex [18] and chronic kidney disease [32], did not show any difference between patients with and without CHF in our study and were thus not included in logistic regression analyses.

## Conclusion

The RV/LV T2-ratio is an easily applicable CMR parameter that does not require a contrast agent and is linked to CHF after STEMI. Especially, a T2-ratio < 60% provides prognostic information for the occurrence of CHF after STEMI, independent of classical risk factors for adverse events after infarction. Patients with a ventricular T2-ratio < 60% experienced CHF earlier and more frequently.

### Limitations

We do acknowledge that this current study bears limitations: to begin, only patients with a Killip class < 3 (at the time of CMR) were included, although especially those patients with a higher Killip class are at higher risk of CHF [33], and thus, it can be assumed that their T2 relaxation times may differ from this current study’s data. Although we have tried to exclude obvious flow artifacts when measuring blood pool T2, we cannot exclude that smaller artifacts might have hampered our measurements. Another point worth discussing is our chosen definition of CHF, which is in accordance with international guidelines; however, a change in definition (e.g., hospitalization and introduction of loop diuretics) would surely have also had an impact on our results. Still, we feel that the used definition represents a sensible and easily objectifiable middle course. Next, all patients were first-time STEMI patients by study protocol; however, the risk of CHF increases in patients with a prior history of myocardial infarction [1]. Lastly, after the last clinical follow-up at 12 months, the telephonic follow-up intervals were quite widely distributed (IQR 456–1470 days); however, CHF after STEMI is known to usually occur within the first few months after acute infarction. Further, telephonic follow-ups always bear the risk of unreliable information given by the respective patient. Although we cannot reliably verify information provided in all cases, we guarantee maximum diligence in the check-up of the yielded information on MACE endpoints. In the end, due to dichotomization, the group with “pathological” low T2-ratios was considerably small (a dichotomization at 67% according to the Youden-index lead to a larger group of 169 patients but yielded less convincing results at logistic regression), which is why larger overall cohorts would probably yield clearer results.

## Supplementary Information

Below is the link to the electronic supplementary material.

ESM1 (DOCX 23.5 KB)

## Data Availability

The datasets used and/or analyzed during the current study are available from the corresponding author on reasonable request.

## Abbreviations

CHF: Congestive heart failure
CI: Confidence interval
CK: Creatine kinase
CMR: Cardiovascular magnetic resonance imaging
CRP: C-reactive protein
EDVi: End-diastolic volume indexed by body surface area
EF: Ejection fraction
ESVi: End-systolic volume indexed by body surface area
GLS: Global longitudinal strain
IMH: Intramyocardial hemorrhage
IQR: Interquartile range
LV: Left ventricle/ventricular
MACE: Major adverse cardiac events
MVO: Microvascular obstruction
NRI: Net reclassification improvement
NT-proBNP: N-terminal pro-brain natriuretic peptide
OR: Odds ratio
PCI: Percutaneous coronary intervention
RV: Right ventricle/ventricular
STEMI: ST-segment elevation myocardial infarction
