# Supplementary material for: Reduced right/left ventricular blood pool T2-ratio predicts congestive heart failure after STEMI
Source: Clin Res Cardiol. 2026 Feb 17;115(10):1819–30. doi: 10.1007/s00392-026-02868-9 (PMC13562305; doi:10.1007/s00392-026-02868-9)
Supplement: Supplementary file 1 [file 392_2026_2868_MOESM1_ESM.docx]

Supplemental Material

| **> Multivariate Analyses (absolute T2 LV)** |  |  |  |
| --- | --- | --- | --- |
| *- Model 1* |  |  |  |
| T2 LV, ms | .9 | .7-1.3 | .705 |
| Diabetes | 3.8 | 1.7-8.9 | **.002** |
| Peak NT-proBNP, ng/l | 1.4 | 1.0-1.9 | .053 |
| IMH | 4.6 | 2.3-9.3 | **<.001** |
| *- Model 2* |  |  |  |
| T2 LV, ms | 1.0 | .8-1.4 | .918 |
| Diabetes | 4.3 | 1.8-10.2 | **.001** |
| Peak NT-proBNP, ng/l | 1.3 | .9-1.9 | .098 |
| Relative Infarct Size, % of Myo Mass | 2.0 | 1.4-2.9 | **<.001** |
|  |  |  |  |
| **> Multivariate Analyses (absolute T2 RV)** |  |  |  |
| *- Model 1* |  |  |  |
| T2 RV, ms | .7 | .5-1.0 | .052 |
| Diabetes | 3.3 | 1.4-7.8 | **.005** |
| Peak NT-proBNP, ng/l | 1.3 | .9-1.8 | .138 |
| IMH | 4.0 | 2.0-8.3 | **<.001** |
| *- Model 2* |  |  |  |
| T2 RV, ms | .8 | .5-1.1 | .105 |
| Diabetes | 3.5 | 1.5-8.6 | **.005** |
| Peak NT-proBNP, ng/l | 1.3 | .9-1.8 | .161 |
| Relative Infarct Size, % of Myo Mass | 1.8 | 1.3-2.7 | **.001** |

**Supplemental Table 1.** Results of logistic regression analyses including absolute values of blood pool T2 for each ventricle. Metric variables were stratified into quartiles.
*CK: creatine kinase, CRP: C-reactive protein, EF: ejection fraction, ESVi: indexed end-systolic volume, GLS: global longitudinal strain, IMH: intramyocardial hemorrhage, LV: left ventricle/ventricular, MVO: microvascular obstruction, NT-proBNP: N-terminal pro-brain natriuretic peptide, RV: right ventricle/ventricular, TnT: troponin T.*
